# Shape Matters: Plant Architecture Affects Chemical Uniformity in Large-Size Medical Cannabis Plants

**DOI:** 10.3390/plants10091834

**Published:** 2021-09-03

**Authors:** Nadav Danziger, Nirit Bernstein

**Affiliations:** Institute of Soil Water and Environmental Sciences, Volcani Center, 68 HaMaccabim Road, P.O. Box 15159, Rishon LeZion 7505101, Israel; nadavdanz@gmail.com

**Keywords:** architecture, cannabis, cannabinoids, pruning, defoliation, standardization, THC, CBD, CBG

## Abstract

Since plant organs sense their environment locally, gradients of micro-climates in the plant shoot may induce spatial variability in the physiological state of the plant tissue and hence secondary metabolism. Therefore, plant architecture, which affects micro-climate in the shoot, may considerably affect the uniformity of cannabinoids in the *Cannabis sativa* plant, which has significant pharmaceutical and economic importance. Variability of micro-climates in plant shoots intensifies with the increase in plant size, largely due to an increase in inter-shoot shading. In this study, we therefore focused on the interplay between shoot architecture and the cannabinoid profile in large cannabis plants, ~2.5 m in height, with the goal to harness architecture modulation for the standardization of cannabinoid concentrations in large plants. We hypothesized that (i) a gradient of light intensity along the plants is accompanied by changes to the cannabinoid profile, and (ii) manipulations of plant architecture that increase light penetration to the plant increase cannabinoid uniformity and yield biomass. To test these hypotheses, we investigated effects of eight plant architecture manipulation treatments involving branch removals, defoliation, and pruning on plant morpho-physiology, inflorescence yield, cannabinoid profile, and uniformity. The results revealed that low cannabinoid concentrations in inflorescences at the bottom of the plants correlate with low light penetration, and that increasing light penetration by defoliation or removal of bottom branches and leaves increases cannabinoid concentrations locally and thereby through spatial uniformity, thus supporting the hypotheses. Taken together, the results reveal that shoot architectural modulation can be utilized to increase cannabinoid standardization in large cannabis plants, and that the cannabinoid profile in an inflorescence is an outcome of exogenous and endogenous factors.

## 1. Introduction

In the booming field of plant-based remedies, cannabis (*Cannabis sativa* L.) is increasingly recognized as a novel medical treatment and a legal recreational drug. The ongoing interest in cannabis originates from effects of the abundant biologically-active secondary metabolites found mainly in the inflorescences, including terpenes, flavonoids, and the uniquely produced cannabinoids [1]. In addition to the known psychoactive effects, cannabis was reported beneficial for the treatment of many ailments including neurological conditions, pain management, and more [2]. The therapeutic effects are attributed to biological interactions between unknown combinations of the secondary metabolites and human receptors [3]. Changes in the chemical profile of the consumed plant material, which is very diverse and includes a wide array of chemotypes, could lead to changes in efficacy.

While the potential for production of a specific secondary metabolite profile in cannabis is determined by the genetic background of the plant [4], the actual levels of the produced metabolites are affected to a large extent by environmental conditions during cultivation, such as mineral nutrition [4,5,6,7], light intensity and light spectrum [8,9], and elicitation of stress conditions [10]. Variability in the chemical profile between inflorescences was observed also along the plant [4,6,7,11]. Furthermore, as plant organs sense their environments locally, differences between micro-climates within the shoot further induce changes in physiology [12] and secondary metabolism [13]. To increase uniformity of the chemical profile within the plant, between plants, and across growing cycles, it is important to understand how different climatic conditions and agricultural practices influence secondary metabolism. This will allow cultivation practices to be harnessed for mitigating chemical variations in the plants by minimizing micro-climatic gradients. The present study aimed to harness plant architecture manipulation for the standardization of the cannabinoid profile in large medical cannabis plants.

Plant architecture has an immense effect on shoot microclimate, affecting light penetration, humidity, and temperature [14]; and in agricultural production systems several practices are used to alter plant architecture, including pruning the main stem/branches, removing branches, and trellising. Another method to affect climate in the canopy that does not affect the plant structure is the complete or partial removal of leaves. The altered climate in the shoot is reported to induce changes in yield quality, such as increased nutritional values due to defoliation in legumes [15] and grapes [16], and quality of pruned melons [17] and bell peppers [18]. Moreover, such architectural alterations can either increase [18] or decrease [15,17] yield quantity.

In cannabis, a single study with industrial hemp cultivars tested effects of pruning the main stem and reported increased seed yield [19]. In “drug type” cannabis, removal of branches was reported to reduce yield biomass and to induce changes in the chemical profile, which varied between cultivars and cannabinoids [20]. It is documented for numerous plant species that changes in plant architecture entail effects on light intensity and spectrum inside the canopy [21]. In cannabis, changes in light intensity and spectrum were found to alter cannabis yield quantity and quality [8,9,22], suggesting a potential of plant architectural manipulation for regulation of localized secondary metabolism and spatial standardization.

Spatial gradients of light intensity in plant canopies intensify with plant height, as the proportion of light reaching the bottom parts of the plant decreases with the increase in the longitudinal penetration distance through the canopy [23]. Therefore, the potential for microclimate-induced alterations of physiological and chemical properties along the plant, is larger in tall-canopy plants compared with smaller plants; and plant architecture manipulation treatments have a potential for mitigating these effects by increasing light penetration to the shoot. In the cannabis production industry, cultivation practices vary, from growing small short plants, usually in controlled growing rooms “indoor”, to larger plants ranging in size in greenhouses or “outdoor”. A considerable portion of the commercial production is based on intensive cultivation of large plants. Spatial gradients in chemical attributes and chemical uniformity in large size cannabis plants have not been studied before, and the potential of plant architectural modulation techniques for increasing intra-plant standardization in such plants is not known.

In the current study, we therefore focused on conditions found in tall plants, reaching ~2.5 m in height at maturity. The hypotheses guiding the study were (i) a gradient in light intensity exists along the plants and is accompanied by changes in plant development and cannabinoid profile; (ii) increased light penetration down the plant by manipulation of the plant architecture will increase yield as well as cannabinoid uniformity throughout the plant. To evaluate these hypotheses, eight architecture manipulation treatments were tested, including the removal of branches and leaves from the bottom of the plants, defoliation, pruning once or twice, and the removal of either primary or secondary branches. Effects on cannabinoid uniformity and plant development were studied.

## 2. Results and Discussion

### 2.1. Canopy Development

**Figure 1:**
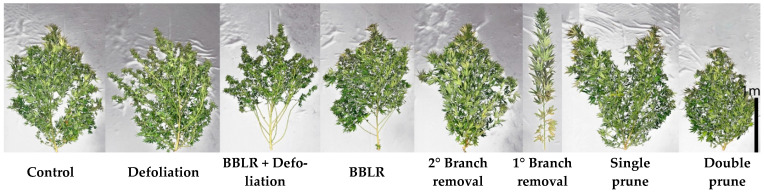
Effect of architecture-manipulation treatments on visual appearance of medical cannabis plants at chemical maturation. BBLR—Removal of leaves and branches from the bottom part of the plant; 1° Branch removal—removal of primary branches; 2° Branch removal—removal of secondary branches. The treatments are detailed in Section 3.2.

Plant shoot growth pattern and shape is commonly manipulated by growers worldwide by numerous techniques including planting density, trellising, plant hormones, and by the physical removal of plant organs. Figure 1 depicts the response of large cannabis plants to seven architecture-manipulating treatments compared to the natural growth habit of the non-treated ("Control") plants. The treatments are detailed in Section 3.2. Plant structure was not altered visually by “Defoliation” and by removal of 2° branches (“2° Branch removal”), and in both "BBLR" (Removal of leaves and branches from the bottom part of the plant) treatments, only the lower part of the canopy differed in form from the non-treated control. Plants of the “Double prune” treatment had similar shape to the “Control” plants, with a shorter stature. Two treatments that visibly altered shoot structure considerably were “1° Branch removal” that is composed of a single elongated straight stem with no branches, and “Single prune”, which induced development of two stem-like branches. These results for the large plants evaluated in the present study correlate with responses we received for two other varieties of medical cannabis under short-plant cultivation [20], with the exception that the shape of the plants of the pruning treatments was less elongated, likely representing genetic differences in the response to alterations of apical dominance, or in growing conditions that promote elongation.

**Figure 2:**
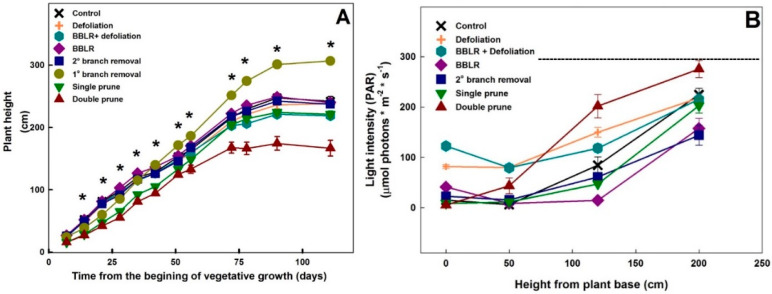
Effect of agricultural techniques that affect plant architecture on height of “drug-type” cannabis plants (**A**), and light intensity at different heights along the plants (**B**). Dashed line in B represents light levels at the greenhouse above the canopy. Data are averages ± SE (*n* = 6). Asterisks above the averages represent significant differences between treatments at a given measurement day, by the Tukey HSD test at α = 0.05.

Plant height varied between treatments (Figure 2A). As early as 7 days after the initiation of the treatments, a statistically significant reduction in height was measured in the pruned plants. Approximately 67 days after the initiation of the treatments, which was 7 days after the transfer to the short-day regime and the second pruning event, the stretched in height of the “Single prune” plants, compensating for the reduction in height imposed by the pruning and the plants reached the height of the “Control” plants.

Architectural manipulations that involve wounding and removal of plant organs alter endogenous developmental programs by affecting apical dominance or sink/source relations and hence also the hormonal profile [24]. Three developmental changes identified in the plants point to involvement of hormonal activity. First, the two semi-main branches developed under the “Single prune” treatment could be attributed to increased amounts of gibberellic acid, which stimulates both plant elongation and inhibition of lateral bud development [24]. Such an increase in active gibberellin production was previously seen in different perennials [25] and could also explain why no reduction in plant height was observed in this treatment. Second, the second pruning event was implemented at the transition to the short day regime, at which time the compact compound inflorescences of cannabis start to develop by restriction of branch elongation and development of short branchlets [26]. It is possible that this developmental shift involves gibberellin catabolism, which could also explain the shorter plants of the “Double prune” treatment, and the increased branching, resulting in the development of “bushier” plants in this treatment. A lack of gibberellin-induced bud dormancy at the switch to short photoperiod might also explain the stimulation of axillary bud growth and development throughout the plant. Third, the “1° Branch removal” treatment caused the main stem to elongate more than in all other treatments. When this treatment was imposed on smaller medical cannabis plants, the same phenotypic response was found in one of the two genotypes studied [20]. Cytokinin is a phytohormone that induces cell division and is highly related to plant branching [27]. The root-derived molecules are transported acropetally in the shoot causing axillary bud development and branching. The removal of branches from the plant removed sink locations, possibly resulting in higher deposition of cytokinin to the apical meristem, inducing enhanced meristem activity and plant elongation.

In cannabis folklore, a “stretching period” of rapid growth is considered to occur at the two weeks following the transition to a short-day photoperiod, prior to the termination of plant elongation and the formation of inflorescences. In the present study, no such “stretching” was seen, but rather a continuation of the pre-short-day growth rate. It is possible that this tale originates from “indoor” growers that conventionally change the cultivation light source from metal-halide (MH) to high pressure sodium (HPS) at the onset of the short photoperiod. These light sources differ in spectrum and intensity. The HPS light spectra is poor in the blue light fraction and rich in the far-red fraction, and both lack of blue light and enrichment in far-red are known elicitors of plant elongation [9]. These results debunk the concept of an endogenous “stretching period” and point at spectral properties as inducers of the growth enhancement.

As the light travels through the plant canopy it is absorbed by both leaves and branches, changing both in intensity and spectrum [23]. To evaluate light abundance within the canopy, light intensity was measured at four different heights, and the results are presented in Figure 2B. Light intensity gradually reduced with the decrease in height towards the bottom of the plants (Figure 2B), and the extent of reduction and the intensity level along the vertical profile differed between treatments. The highest intensities at the plant base were obtained for both defoliation treatments, demonstrating the potential of defoliation for reducing micro-climate gradients.

Surprisingly, both “BBLR” treatments had higher light intensity at the bottom of the plant than at the height of 50 cm aboveground (Figure 2B). This is likely caused by horizontal introduction of light to this part of the plants, which is sparse in vegetation due to the treatment, by reflectance from side plants, or by light penetrating from the space between plant rows.

Light intensity at the location 50 cm aboveground was similar for both “Defoliation” treatments (Figure 2B). This indicates that light absorbed by branches and inflorescences was similar in both treatments, and that in spite of the removal of bottom branches, shoot density was similar in both treatments, as will also be demonstrated by the effects of the treatments on the plant biomass. The difference in light intensity at the plant base between the “BBLR + defoliation” and the “Defoliation” treatments was similar to the difference between the “BBLR” and “Control” treatments and was small in both cases, ranging from 25 to 41 μmol m^−2^ s^−1^. This quantifies the amount of light reflected from the sides at the bottom of the plot to be between 25 and 41 μmol m^−2^ s^−1^.

Light penetration to the shoot affects plant development in numerous ways. First, increased light intensity at the lower area of the shoot goes hand-in-hand with a warmer and drier micro-climate [14]. Both increased light exposure and reduced relative humidity promote transpiration and photosynthesis rate [28], supporting accelerated growth. This was demonstrated in numerous cropping systems. In eggplant (*Solanum melongena*), for example, increased photosynthesis was recorded in plants pruned to increase light penetration to the canopy, resulting in increased content of carbon assimilates [29]. Such improved plant function is desired in intensive agriculture, and plant canopy manipulation is therefore often used to increase light penetration. In *Camellia oleifera* Abel., an open-center shape increased light penetration and temperature at the bottom of the canopy and reduced relative humidity. This altered microclimate increased seed yield and oil contents at the lower part of the canopy compared to a round, closed canopy shape [14]. In apple trees, light penetration positively correlated with flower bud density, fruit yield, fruit skin color, soluble solids, and fruit firmness [30]. With the potential for improved chemical composition due to increased light penetration, the cannabinoid profile of the plants was examined next.

### 2.2. Chemical Response

**Figure 3:**
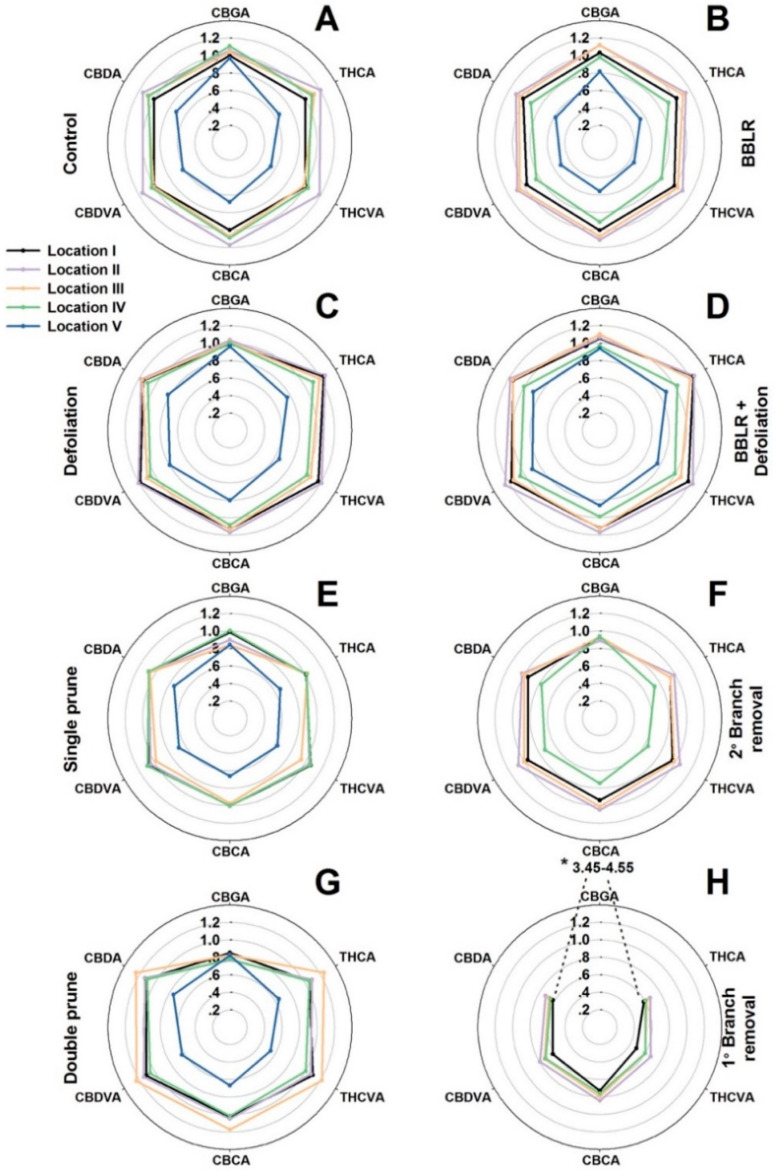
Relative concentrations of six cannabinoids at five locations along the plant, as affected by plant architecture-manipulation treatments: “Control” (**A**), “Defoliation” (**B**), “Defoliation + BBLR” (**C**), “BBLR” (**D**), “2° Branch removal” (**E**), “1° Branch removal” (**F**), “Single prune” (**G**), “Double prune” (**H**). Presented results for each cannabinoid are ratios relative to the concentration at the apical inflorescence on the main stem (location I) of the “Control” plants. CBGA levels in the “1° Branch removal” treatment were 3.45–4.55 (out of the scale of the unified spider charts). Data are means of six replicated plants per treatment (*n* = 6). “BBLR”—removal of leaves and branches from the bottom part of the plant. The treatments are detailed in Section 3.2.

The difference between cultivation of large vs. small plants entails differences in plant physiology and larger variations in micro-climate conditions along the plant [31,32]. A longer distance between the shoot apex and the plant base entails larger hormonal and micro-environment gradients and lower values at the plant base [31]. In addition, in taller plants, more plant organs (at the center and bottom of the plants) suffer from shading, as more leaves and branches above absorb light [33]. As cannabis inflorescence development is affected by both endogenous and exogenous factors, it is likely that bigger plants will suffer from increased spatial variability compared with smaller plants. To compare variations along the plant and between treatments, the average concentration of each cannabinoid in each sampled location was compared to the concentration in the primary, apical inflorescence (location I) of the “Control” treatment (Figure 3). In Figure 3, the further a data point is from the center, the higher is the concentration of the cannabinoid in the specific location compared to the concentration at location I of the control plants. The absolute values represented by this figure can be found in Supplemental Figures S1–S5.

In the major cannabinoid biosynthetic pathway, cannabigerolic acid (CBGA) is the first cannabinoid formed, which serves as a precursor for an enzymatic catalyzed biosynthesis of the primary cannabinoids ∆^9^-tetrahydrocannabinolic acid (THCA), cannabidiolic acid (CBDA), and cannabichromenic acid (CBCA(. Similarly, in a parallel minor pathway, ∆^9^-tetrahydrocannabivarinic acid (THCVA), cannabidivarinic acid (CBDVA), and cannabichromevarinic acid (CBCVA) are formed from cannabigerovarinic acid (CBGVA). The concentration of CBGA was considerably affected by the treatments and demonstrated a treatment-dependent response. It was highest in the “1° Branch removal” treatment (Figure 3H) and reached a level 3.7–4.6-times higher than in the “Control” plants (Figure 3A). However, in all other treatments and locations, except for location II and III in both “BBLR” treatments (Figure 3B,D), CBGA concentration was lower than the “Control” concentration. Since CBGA is the precursor of all other cannabinoids, its concentration in the plant is dynamic and reflects the net activities of its biosynthesis and further transformation down the cannabinoid pathways. A question arises as to whether the high level of CBGA in the “1° Branch removal” plants is due to intensified biosynthesis, or rather a reduction in its enzymatic transformation to other cannabinoids. As the concentrations of all other cannabinoids in the “1° Branch removal” treatment were reduced considerably (by 25–40%) compared to the “Control”, it implies that the CBGA “enrichment” in this treatment is a result of inhibition of metabolic activity down the cannabinoid pathway.

Contrary to the common belief in the cannabis industry that consider the cannabinoid concentrations of the primary inflorescence (the “cola”) to be highest in the plant, we report here that for most treatments (e.g., “Control”, “2° Branch removal”, “Double prune”, and both “BBLR” treatments), the concentrations of most cannabinoids were higher in locations II and III than in the apical meristem of location I. Furthermore, in both the “Control” and “Single prune” plants, location IV also had higher concentrations than location I. This result is supported by results of Danziger and Bernstein [20] that reported as well that cannabinoid concentrations in the primary inflorescence were not always the highest in the plant. As cannabinoid concentrations in an inflorescence are affected by exogenous (environmental) factors, as well as endogenous developmental/location effects, the difference between treatments in this phenomenon should be evaluated taking into consideration both these aspects. From a microclimate standpoint, locations I, II, and IV are at the top of the plant and are highly exposed to sunlight with little to no shading by other plants or branches. Location III on the other hand is also highly exposed but could be shaded by adjacent plants within the row or by plants from parallel rows at certain hours of the photoperiod. Similar to light intensity, air circulation as well is expected to be prevalent in these locations, maintaining similar temperature and humidity at locations I and II and to a lesser extent at locations III and IV. Hence, environmental variations alone cannot explain the identified variations in concentrations.

As micro-climate was partially contradictive as the cause of the observed variation in concentrations, developmental/location effects should be considered. Within the plant, changes in the timing of chemical maturation between inflorescences of different developmental orders may have been reflected as a variability in the chemical profile. A possible explanation is that the primary apical meristem of location I, as the dominant growing zone of the plant, is slower to transition to the reproductive phase, or slower to cease inflorescence growth, thus delaying the maturation of the cannabinoid chemical profile, resulting in lower abundance of secondary metabolites at the time of harvest.

In all treatments, location V (IV in the “2° Branch removal” treatment) was significantly lower down the plant than the remaining locations. Even though at all other locations some variation in light intensity caused little to no effect on secondary metabolism, the cannabinoid concentrations at location V were reduced considerably in all treatments by up to 40% (Figure 3). As at this location light levels were very low, light appears to be a limiting factor for secondary metabolism. The treatments that suffered least from light scarcity at the location of the bottom-most inflorescence were both “Defoliation” treatments, which also had the smallest decline in cannabinoids in this location. The ability of these treatments to mitigate cannabinoids loss, even though the defoliating was imposed only three weeks before harvest, indicates the high importance of light throughout the process up to chemical maturation.

There was a spatial difference between the ratios of THCA:THCVA and CBDA:CBDVA. For example, in the “BBLR + Defoliation” treatment, the relative increase in CBDVA between locations II and IV was higher than the relative increase in CBDA, but the increase in THCA was higher than THCVA. The biosynthetic pathway of these cannabinoids is intertwined, as both THCA and CBDA originate from the CBGA precursor and both THCVA and CBDVA originate from CBGVA. However, both THCVA and THCA are synthesized by the tetrahydrocannabinolic acid synthase (THCA synthase) enzyme, while CBDA and CBDVA are synthesized by the cannabidiolic acid synthase (CBDA synthase) enzyme [34]. The difference in ratios between the two pairs (THCA:THCVA and CBDVA:CBDA) suggests differences in substrate selectivity between the enzymes, as well as some environmental effects on their activity or biosynthesis, which should be further explored. Environmental conditions such as light are known to effect some enzymes’ activities [35] and could explain the previously-described reduction of metabolites in location V.

Secondary metabolism is influenced by environmental conditions such as light [36], temperature, humidity, mineral nutrition, and water availability [37], as well as intrinsic factors such as tissue age and location [38]. Several studies examined the influence of light quality on chemical quality in cannabis and identified variations in the cannabinoid profile [8,9,39], which may explain in part the considerable reduction in cannabinoid content at the bottom of the plant. According to Ma et al. [40], flavonoid concentrations decreased with the reduction in light intensity in *Anoectochilus formosanus*, and similarly, a decrease in phenolic compounds with lower daily light integrals was detected in sweet basil (*Ocimum basilicum*) [41]. The reduced cannabinoid levels can be partially explained by light quality alteration and lowered light intensity at the bottom of the plants.

**Figure 4:**
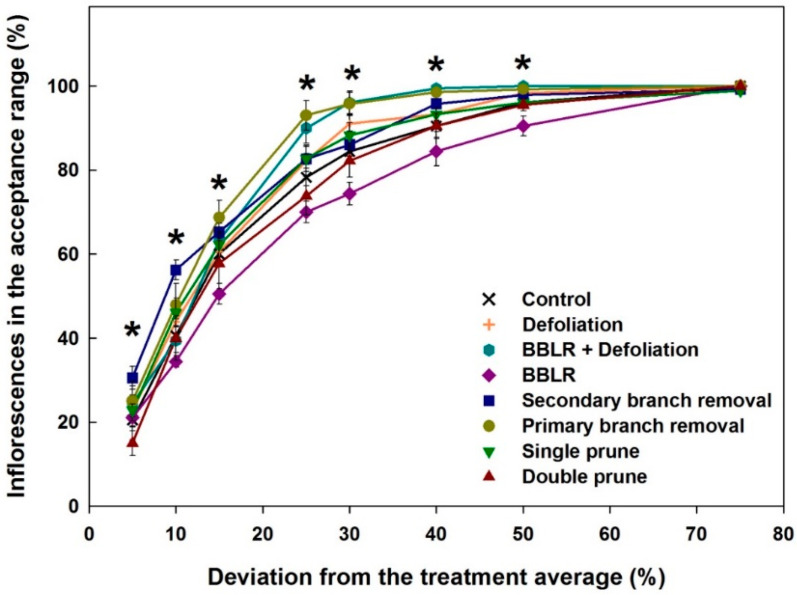
“Plant uniformity score” of the cannabinoid profile under the architecture treatments tested. Results are averages at the acceptance range of 5–75% deviation from the treatment average. Asterisks above the means represent significant differences between treatments at a specific deviation value, by Tukey HSD test at α = 0.05.

The results in Figure 3 reveal variability in concentrations of cannabinoids between locations in the plant in all treatments. To evaluate if the investigated treatments affected the extent of variability and have the potential for standardization of the chemical potential, we analyzed the effect of the treatments on uniformity of the chemical profile within the plant. A new score, “Plant Uniformity score” (PUS) that was recently developed by us [20] was applied for the analysis. The “Plant Uniformity score” (PUS) integrates the variabilities of individual cannabinoids to generate an integrated plant uniformity value. This score allows to calculate the percentage of inflorescences in a treatment which their chemical profile deviates from the treatment average by a pre-defined percentage, for example, a deviation by more than ±5%, 15%, or 30% from the treatment average, etc. Figure 4 presents the plant uniformity score starting at a strict 5% deviation restriction, and up to an inclusive 75% deviation limit. High uniformity score is achieved by chemically similar inflorescences regardless of the treatment average concentration of each cannabinoid. The analysis revealed a few treatment-related effects: (i) All treatments except “Double prune” and “BBLR” improved chemical uniformity compared to the “Control”; (ii) BBLR was the least uniform treatment in the 10–50% range; (iii) in the strict 5–10% deviation range, "2° Branch removal” was the most uniform. but for the more accepting terms, “1° Branch removal” was more uniform; (iv) “BBLR + Defoliation” was the most uniform treatment (when excluding the branch removal treatments); (v) at all treatments, a “perfect score” (>95 uniformity) was achieved in the 50% deviation rate except for “BBLR”, which reached this goal only at the 75% level of deviation restriction. These results are similar to results reported by Danziger and Bernstein [20] for smaller cannabis plants. In both studies, a perfect score was reached at the 50% restriction level by all treatments except for “1° Branch removal” and “2° Branch removal”, and a general increase in uniformity was achieved by most treatments compared to the “Control". This identified similarity between plants of different genetic background and size suggests that a maximum of 50% chemical variation from the average concentrations is a universal characteristic expected for cannabis plants regardless of their size. On the other hand, under a more restrictive levels of variance, the smaller plants grown by Danziger and Bernstein [20] had a plant uniformity score higher by 5–15 points, demonstrating that a higher uniformity was achieved by cultivating smaller plants, though this should be tested under similar growing conditions and varieties.

The chronic lack of light at the base of the plant that we found to be inherent in the cannabis plants, induces chemical variation, and most likely also reduces floral yield. This restriction of light availability down the canopy could be mitigated by local supplementation of light by sub-canopy or intra-canopy illumination. Under such illumination, the lighting systems give light to the middle and bottom canopy, which can lead to higher whole plant photosynthesis and better crop productivity. Many studies have shown that supplemental lighting within the canopy can enhance yield of crop plants [42,43,44]. A previous study assessed the possibility to increase cannabis yield by sub-canopy LED lights, and the results showed a 5% increase in THCA + THC concentrations, but not other cannabinoids, as well as a 13–17% increase in inflorescence yield [22]. Effects on chemical standardization were not evaluated. The supplemented light within the canopy could be used not only to increase light intensity, but also to compensate for the change in spectral quality as it passes through leaf tissue [23].

**Figure 5:**
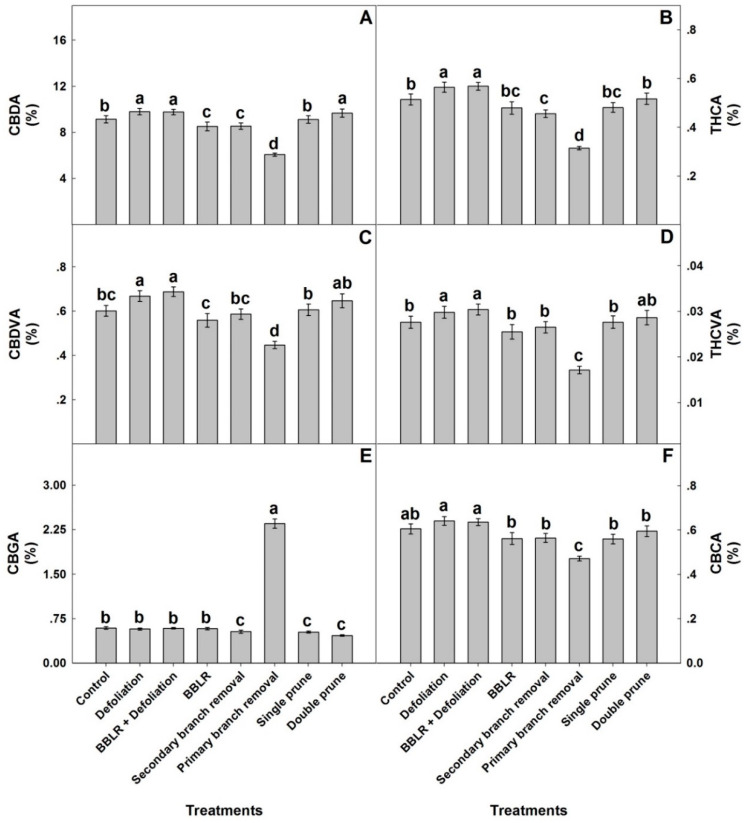
Effect of architecture manipulations on average cannabinoid concentrations in medical cannabis plants: CBDA (**A**), THCA (**B**), CBDVA (**C**), THCVA (**D**), CBGA (**E**), and CBCA (**F**). Data are means ± SE (*n* = 6). Letters above the bars represent significant differences between treatments by Tukey HSD test at α = 0.05.

To compare responses of individual cannabinoids to the architecture manipulation treatments, the plant average concentrations of each cannabinoid are compared in Figure 5. The plant average concentrations of most identified cannabinoids (CBDA, THCA, CBDVA, THCVA, and CBCA) had a similar yet not identical response to the plant architecture treatments, while the response of CBGA differed considerably. A plant-average concentration was affected by all in-planta variations, and an important finding is that it was similar to the concentration at the primary inflorescence at location I the “cola”), which is the industry standard, and therefore demonstrated less variability between treatments than was found for some of the individual locations. The plant average concentrations are the relevant concentrations for industrial production of extraction-based products, whereas concentrations at individual locations and their in-planta variability are crucial for direct consumption of dry inflorescences by smoking and vaporizing. Results for all individual locations can be found in Supplemental Figures S1–S5.

CBGA levels of the plant average concentrations were highest in the “1° Branch removal” plants, reaching 2.38% of the inflorescence dry weight, while in all other treatments the concentration range was 0.45–0.6% (Figure 5). Both pruning treatments and “2° Branch removal” had statistically lower CBGA concentrations than the “Control”, “BBLR”, and both defoliation treatments. For all other identified cannabinoids (except CBCA), both defoliation treatments had significantly higher concentrations than the “Control”, while “1° Branch removal” was always lower. In the cannabis plant, CBGA is synthesized from olivetolic acid and geranyl diphosphate. It is the precursor for most cannabinoids and is converted in the plant by CBCA synthase, CBDA synthase, and THCA synthase to the three primary cannabinoids, namely CBCA, CBDA, and THCA, respectively. The primary cannabinoids are then converted downstream to numerous cannabinoid families [1]. The considerably higher concentrations of CBGA in the “1° Branch removal” treatment may therefore result from reduced biosynthesis of CBGA or from inhibited metabolic conversion of CBGA to the three primary cannabinoids. The results, which demonstrated lower concentrations of the primary cannabinoids in the “1° Branch removal” treatment compared to all other treatments, point at inhibition of the conversion of GBGA to the three primary cannabinoids in this treatment, likely due to restricted energy availability in these suppressed plants.

In general, the plant average concentrations of all cannabinoids except CBGA followed the trend of “Defoliation” = “BBLR + Defoliation” > “Double prune” > “Control” = “Single prune” > “BBLR” = “2° Branch removal” > “1° Branch removal”.

### 2.3. Inflorescence and Cannabinoid Yield

**Figure 6:**
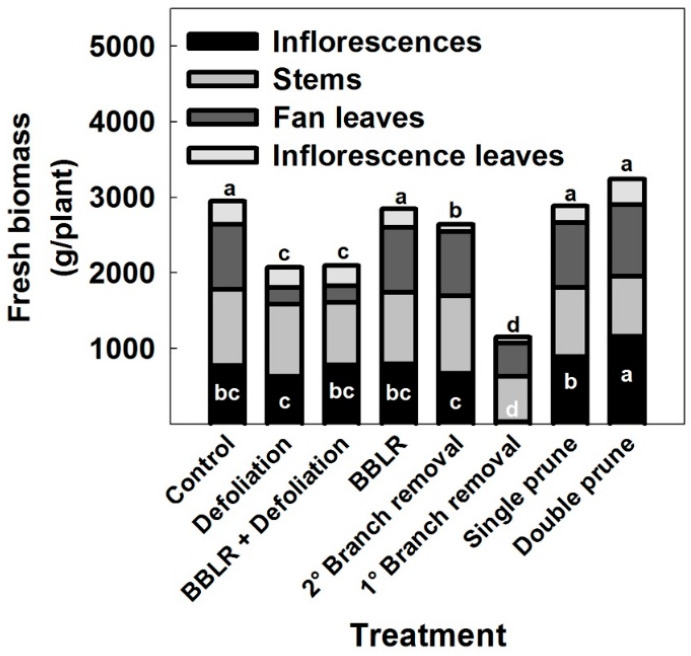
Biomass of inflorescences, stems, fan leaves, and inflorescence leaves in “drug-type” medical cannabis, as is affected by plant architecture manipulations. Lowercase letters inside and above the bars represent significant differences in inflorescences biomass and in total shoot biomass, respectively, between treatments by Tukey HSD test at α = 0.05. Presented data are averages (*n* = 6).

In many crops, shoot architectural manipulations induce not only morphological changes but also affect yield. In cannabis, yield could be described as either inflorescence biomass (per plant or cultivation area), or as cannabinoid production (per plant or cultivation area). In some industrial cannabis production schemes, vegetative plant biomass is also referred to as yield, as it can be utilized for example in the textile or construction industries [45]. Fresh biomass accumulation by different plant organs in the medical cannabis plants is presented in Figure 6. Inflorescence yield production per plant was affected by the treatments. Plants of the “Double prune” treatment produced higher yield (*p* < 0.05) than the “Control”, and the “1° Branch removal” plants had lower yield than the control (*p* < 0.05). All other treatments did not affect significantly yield biomes (Figure 6), demonstrating developmental plasticity of the reproductive growth in response to the invasive changes in shoot architecture.

The “1° Branch removal” treatment reduced inflorescence yield considerably, by up to 99%. This dramatic reduction in yield is a result of the absence of active meristems in the plants due to the removal of branches and branchlets by the severe pruning, which prevented inflorescence development along the stem, as well as by a reduction in photosynthetic leaf area per plant, which reduced energy production. This result is supported by a similar decline in inflorescence yield that was reported for smaller cannabis plant cultivation [20].

As both inflorescence yield and chemical profile were altered by the architecture manipulation treatments, it should not be surprising that the cannabinoid yields per plant were also affected. In Table 1 the results of cannabinoid yield (g/plant) are presented. In spite of the four-times higher concentration of CBGA in the “1° Branch removal” plants compared with all other treatments (Figure 5), CBGA production per plant in this treatment was significantly lower (*p* < 0.05; Table 1). Dramatically lower production of cannabinoids per plant in this treatment was apparent also for all other identified cannabinoids, which accumulated to only ~3% of the cannabinoid yield produced by other treatments in terms of g/plant.

For all cannabinoids, higher yields (g/plant) were produced by the “Double prune” plants compared to all other treatments (for CBGA the difference was not statistically different; Table 1). The “Defoliation” and both “BBLR” treatments, as well as the “2° Branch removal” and “Single prune” treatments did not affect significantly (*p* > 0.05) cannabinoid yields (as compared with the non-treated control; Table 1). The highest cannabinoid yield was achieved by the increased inflorescence yield in the “Double prune” treatment, more so than by the increase in cannabinoid concentrations in the defoliation treatments. This demonstrates that in spite of the expected diluting effect due to growth, the additional inflorescence tissue benefited whole plant cannabinoid production more so than the induced increases in tissue concentrations. This conclusion can be utilized for optimization of production schemes for specific production goals.

The increase in inflorescence biomass in the “Double prune” treatment compared to the control contributed more to the cannabinoid yield than the increase in cannabinoid concentrations at the “bottom” inflorescences by light penetration in the defoliation treatments. In different medicinal plants, the essential oil is the desired component, and efforts to increase its concentration as well as total yield include optimization of light intensity as well as moderate water stress. For rosemary (*Rosmarinus officinalis* L.), higher light intensity with no water stress resulted in increased essential oil yield in spite of the reduction in tissue concentration. On the other hand, decreased light intensity coupled with moderate water stress resulted in a yield similar to the high light intensity treatment due to significantly higher essential oil concentration [46]. *Eucalyptus citriodora*, on the other hand, produced less essential oil under full sun, probably due to light-induced stress [47], while Chaste tree (*Vitex agnus-castus* L.) and sweet basil (*Ocimum basilicum*) responded to decreased light intensity and decreased water availability with decreased essential oil production [48,49]. In most reported studies, the change in biomass was more influential on total secondary metabolites yield than changes in metabolites concentrations, most likely because the range of changes of secondary metabolite concentrations is smaller than effects on biomass accumulation. It is thus suggested that maximizing inflorescence yield weight should be the first line strategy in cannabis, to be fine-tuned by optimization of cannabinoid concentrations. It should however be considered that the longitudinal light gradient down the plant has a considerable effect on cannabinoid uniformity, with fluctuations of up to 50% between locations.

## 3. Materials and Methods

### 3.1. Plant Material and Growing Conditions

The medical cannabis (*Cannabis sativa* L.) cultivar “Topaz” (BOL Pharma, Revadim, Israel) was used for the study. It is a type III cultivar containing high CDB (8–16%) and low THC (<1%) levels. The experiment was conducted in a certified commercial cannabis farm (BOL Pharma, Revadim, Israel) in a naturally lit greenhouse with photoperiodic light supplementation. Plants were developed from cuttings in a coconut fiber mixture (plugs, Jiffy international AS, Kristiansand, Norway). The rooted cuttings were planted in 13 L pots, 1 plant per pot, in a peat moss mixture (Kekkila-BVB, De Lier, Netherlands) at the density of 1 plants/m^2^. Uniform plants were randomly divided into eight groups of six plants each, and the groups were randomly assigned a treatment. At the vegetative growth stage, the plants were cultivated under a long photoperiod, of 24/0 h of light/darkness, and photoperiodic illumination was supplemented by fluorescent lights. To generate large plants at maturation, the plants were grown for an extended period of 63 days at the vegetative phase, pre-flowering under long photoperiod. After 63 days of vegetative growth, the plants were transferred to a short photoperiod of 12 h to induce flowering. Fertilizers were supplied by fertigation, i.e., dissolved in the irrigation solution at each irrigation event (“Shefer” 5-1.5-8, ICL, Haifa, Israel). Irrigation was supplied via 1.2 L/h discharge-regulated drippers (Plastro Gvat, Israel), four drippers per pot. The volume of irrigation water in each irrigation event was set to allow ~30% of drainage, and it increased throughout plant development up to 3 L/pot/day. The experiment was terminated 111 days after planting, 58 days after the transition to the short photoperiod, at the maturation stage accepted for commercial harvesting.

### 3.2. Experimental Treatments and Design

Eight architectural manipulation treatments were evaluated: (i) a non-treated control (Control); (ii) defoliation 3 weeks prior to harvest; (Defoliation); (iii) removal of the branches and leaves from the lower 1/3 part of the plant at the transition to the short-day (we named this treatment “Bottom branches and leaves removal” (BBLR); this treatment is also known as “Lollipoping” in the cannabis industry jargon); (iv) BBLR + defoliation; (v) removal of all branches off the main stem throughout the growing period (1° Branch removal); (vi) removal of all secondary branches from the main branches throughout the growing period (2° Branch removal); (vii) pruning the rooted cuttings at the day of transplanting to the experimental pots, leaving six primary branches (Single prune); (viii) pruning the plants twice, at the duration of the Single prune and a second time at the transition to the short-day (Double prune). The experiment was conducted in a random experimental design, with six replicates. Replicated groups for each treatment was arranged in 3 rows, out of which a plant from the central row was sampled.

### 3.3. Plant Growth, Biomass Accumulation, PAR, and Yield

The height of each plant was measured biweekly from the plant base to the top of the apical meristem of the main stem (in the pruning treatments the height of the highest branch was measured). At the final harvest, biomass of inflorescences, stems, and fan leaves were measured for each plant individually with an industrial “Mierav 4000" scale (Shekel, Beit Keshet, Israel). Inflorescences were then trimmed by an industrial trimmer for removal of protruding inflorescence leaves as is conventionally practiced in the cannabis industry (Keirton Inc. Ferndale, WA, USA), and the trimmed inflorescences were weighted again for the calculation of the trimmed inflorescence leaves biomass. Inflorescence yield was evaluated following drying to the conventional industry standard of 15% water in the tissue. The cannabinoid yield was calculated by multiplying the average concentration of each cannabinoid in the plant by the inflorescence dry weight yield biomass of the plant.

Photosynthetic active radiation (PAR) was measured at four locations in each plot, at four heights along the plant (0, 0.5, 1.2, and 2 m from the plant base) using an Apogee quantum sensor MQ-500 (Apogee Instruments, Logan, UT, USA).

### 3.4. Cannabinoid Analyses

**Figure 7:**
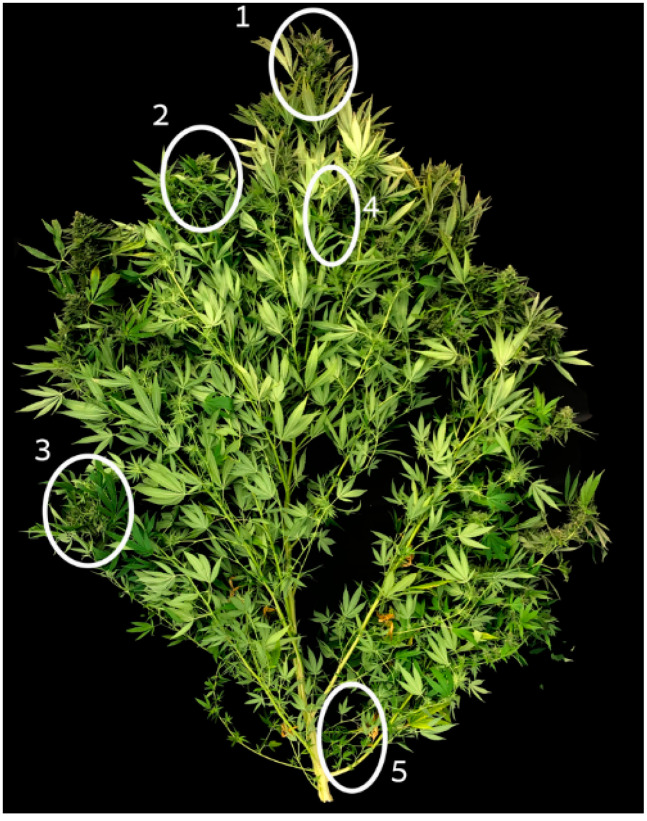
Inflorescences sampling locations 1–5 along the plant. Image of a control pant.

For the evaluation of the effect of the treatments on the standardization of the cannabinoid profile in the plant, inflorescences from 5 locations along the plants were analyzed for cannabinoid concentrations. The locations sampled (see Figure 7) were (1) the apical inflorescence on the main stem (for both pruning treatments, the top-most apical inflorescence on the plant was sampled); (2) apical inflorescence from a 1° branch, from the top 1/3 of the plant (the 4th branch from the plant top; for the double-prune treatment, the top most inflorescence of the 2nd or 3rd branch from the top was sampled); (3) apical inflorescence of a 1° branch from the bottom 1/3 of the plant (4th branch from the plant base; for the double-prune treatment, the top most inflorescence of the branch was sampled); (4) inflorescence close to the stem from an upper branch (2nd branch from the top of the plant); and (5) inflorescence close to the stem from the bottom of the plant—the smallest and least developed inflorescences were sampled (from the 1st branch from the plant base; the 2° Branch removal treatment was not sampled for this location). For the “1° Branch removal” treatment, which developed a single inflorescence at the top of the plant, we evaluated spatial standardization within the inflorescent by sampling four locations: 1—top of the inflorescence; 2 and 3: middle sections from two opposite sides of the inflorescence; and 4—the bottom part of the inflorescence. Trimmed inflorescences were dried at 24 °C and 55% air humidity for 14 days, in an environmentally controlled chamber, in the dark. The analyses were conducted for 6 replicated plants per treatment, for one inflorescence per location per plant, following the experimental design.

For the cannabinoid analysis, the top of the dried inflorescences was ground using a plastic handheld herb grinder (manufacturer unknown). Fifty milligrams of the ground tissue were placed in a 20 mL glass vials, 10 mL ethanol were added to each vial, and the vials were shaken in a reciprocal shaker for 1 h at room temperature. The extracts were filtered through a polyvinylidene difluoride (PVDF) membrane filters of 0.22 µm pore size (Bar-Naor ltd, Ramat Gan, Israel). Cannabinoid concentrations in the filtered extracts were analyzed using a Jasco 2000 Plus series high performance liquid chromatography (HPLC) system, which consisted of a quaternary pump, auto sampler, column compartment, and photodiode array (PDA) detector (Jasco, Tokyo, Japan). The detection was conducted in the spectrum mode. Chromatographic separations were carried out using a Luna Omega 3 µm Polar C18 column (Phenomenex, Torrance, CA, USA) employing 75:25 (*v*/*v*) acetonitrile:water with 0.1% (*v*/*v*) formic acid, at the isocratic mode, with a flow rate of 1.0 mL/min. Quantification of cannabinoid concentrations were based on pure analytical standards purchased from Sigma-Aldrich (Germany): cannabichromenic acid (CBCA(, cannabichromene (CBC), cannabichromevarin (CBCV), ∆^9^-tetrahydrocannabivarinic acid (THCVA), cannabigerolic acid (CBGA), cannabigerol (CBG), cannabinol (CBN), cannabinolic acid (CBNA), cannabidiol (CBD), cannabidiolic acid (CBDA), cannabicyclol (CBL), cannabidivarin CBDV, and cannabidivarinic acid (CBDVA); Cayman chemical company (Ann Arbor, MI, USA): cannabicitran (CBT); and Restek (Pennsylvania, USA): ∆^9^-tetrahydrocannabivarin (THCV), ∆^9^-tetrahydrocannabinolic acid (THCA (THCA-A), ∆^9^-tetrahydrocannabinol (THC), and ∆^8^-tetrahydrocannabinol (Δ^8^-THC). R^2^ values for linear regressions of the calibrations curves of all cannabinoid standards were >0.994 [7]. Concentrations of CBDV, CBG, THCV, CBC, CBN, CBNA, Δ^8^-THC, CBL, CBT, and THC were lower than the detection limits.

### 3.5. Evaluation of Spatial Uniformity (Standardization) of the Cannabinoid Profile in the Plant

Two scores were developed for the evaluation of the treatment effects on the variability of the cannabinoid profile in the plant. “Cannabinoid Uniformity score” (CUS) is a measure of the variability within a treatment of a single cannabinoid. It was defined as the percentage of inflorescences that their concentration of the specific cannabinoid deviated by more than ±15% from the treatment average concentration for this cannabinoid (Equation (1)). The average concentration of a cannabinoid per treatment was calculated from the independent analytical results of 25–30 samples per treatment (presented in Figures S1–S5 Supplemental). “Plant Uniformity score” (PUS) is a measure of the integrated variabilities of all the cannabinoids identified within a treatment. It was calculated by averaging all CVSs per treatment (Equation (2)). The higher the score, the less variable the treatment.

(1) $$\mathrm{Cannabinoid}\;\mathrm{Variation}\;\mathrm{Score}\left(\mathrm{CUS}\right)=\frac{\mathrm{No}.\;\mathrm{of}\;\mathrm{samples}\;\mathrm{with}\;\mathrm{cannabinoid}\;\mathrm{conc}.\;<\pm 15\%\;\mathrm{from}\;\mathrm{the}\;\mathrm{treatment}\;\mathrm{average}}{\mathrm{No}.\;\mathrm{of}\;\mathrm{samples}\;\mathrm{that}\;\mathrm{contained}\;\mathrm{the}\;\mathrm{cannabinoid}}*100$$

(2) $$\mathrm{Plant}\;\mathrm{Variation}\;\mathrm{Score}\;\left(\mathrm{PUS}\right)\;=\;\frac{\sum \mathrm{CUS}}{\mathrm{number}\;\mathrm{of}\;\mathrm{cannabinoids}}$$

### 3.6. Statistical Analysis

The statistical analysis was performed with the Jump software (version 9, SAS 2015, Cary, NC, USA). The data were subjected to one-way ANOVA (α < 0.05) followed by Tukey’s HSD post-hoc test for separation of means. The data met the assumption of homogeneity of variances. Comparison of relevant means was conducted using the Tukey LSD test at a 5% level of significance.

## 4. Conclusions

Cultivation of large plants enables growers to increase cannabis yield, but information has been missing on the properties and chemical uniformity of such yield. Since effects of plant size and plant architecture on the microclimate in the shoot are intertwined, we focused on the interrelations between architectural manipulation treatments and spatial standardization of the cannabinoid profile. This was aimed at optimization of plant structural manipulations for improvement of yield quantity and chemical quality. The results revealed that pruning the plants twice during cultivation was the optimal practice for increasing yield, and other treatments decreased or did not affect yield quantity. While some changes to the chemical profile were induced by the treatments and they generally followed a similar pattern of “Defoliation” = “BBLR + Defoliation” > “Double prune” > “Control” = “Single prune” > “BBLR” = “2° Branch removal > “1° Branch removal”, effects of these chemical changes on overall cannabinoid production per plant were secondary to effects of floral yield biomass. Therefore, architecture manipulation can be utilized to increase yield biomass and standardization, but the cannabinoid yield should be addressed by other means. A considerable reduction of light interception down the shoot was observed, and the extent of spatial standardization of the cannabinoid profile correlated with the effect of plant architecture on light penetration to lower parts of the canopy. This suggests that low light availability at the bottom of the plant is a powerful inducer for reduction of spatial chemical standardization. The increased yield achieved by growing large plants comes at the cost of low chemical uniformity in the plant. These results are instrumental for directing development of optimized cultivation protocols for the cannabis industry, for ensuring high quality medical product for patients.

## Figures and Tables

**Table 1:** Effect of architecture-manipulation treatments on cannabinoid yield per plant in “drug-type” cannabis. Different superscript letters in a column represent significant differences between treatments by Tukey HSD test at α = 0.05. Data are averages ± SE (*n* = 6).

Architecture Manipulation Treatments	CBGA (g/plant)	CBDA (g/plant)	CBDVA (g/plant)	THCA (g/plant)	THCVA (g/plant)	CBCA (g/plant)
**Control**	0.917 ± 0.06 ^ab^	14.099 ± 0.95 ^bc^	0.928 ± 0.06 ^b^	0.794 ± 0.05 ^bc^	0.043 ± 0 ^b^	0.933 ± 0.06 ^b^
**Defoliation**	0.783 ± 0.08 ^b^	13.364 ± 1.43 ^bc^	0.911 ± 0.1 ^b^	0.769 ± 0.08 ^bc^	0.041 ± 0 ^b^	0.873 ± 0.09 ^b^
**^1^ BBLR + Defoliation**	0.921 ± 0.09 ^ab^	15.287 ± 1.42 ^bc^	1.076 ± 0.1 ^b^	0.89 ± 0.08 ^b^	0.048 ± 0 ^b^	0.994 ± 0.09 ^b^
**BBLR**	0.927 ± 0.05 ^ab^	13.584 ± 0.74 ^bc^	0.891 ± 0.05 ^b^	0.765 ± 0.04 ^bc^	0.041 ± 0 ^b^	0.895 ± 0.05 ^b^
**2° Branch removal**	0.711 ± 0.03 ^b^	11.453 ± 0.52 ^c^	0.786 ± 0.04 ^b^	0.611 ± 0.03 ^c^	0.036 ± 0 ^b^	0.756 ± 0.03 ^b^
**1° Branch removal**	0.146 ± 0.01 ^c^	0.377 ± 0.03 ^d^	0.028 ± 0 ^c^	0.02 ± 0 ^d^	0.001 ± 0 ^c^	0.029 ± 0 ^c^
**Single prune**	0.931 ± 0.04 ^ab^	16.194 ± 0.7 ^b^	1.076 ± 0.05 ^b^	0.855 ± 0.04 ^bc^	0.049 ± 0 ^b^	0.992 ± 0.04 ^b^
**Double prune**	1.079 ± 0.08 ^a^	22.446 ± 1.67 ^a^	1.5 ± 0.11 ^a^	1.199 ± 0.09 ^a^	0.066 ± 0 ^a^	1.378 ± 0.1 ^a^

^1^ BBLR = removal of leaves and branches from the bottom of the plants.

## Data Availability

The data are included in the manuscript.

## Supplementary Materials

The following Supplementary data are available online at https://www.mdpi.com/article/10.3390/plants10091834/s1: Figure S1: Impact of plant architecture treatments on cannabinoid concentrations in the apical primary inflorescence (location 1); Figure S2: Impact of plant architecture treatments on cannabinoid concentrations at the apical inflorescence of the highest branch on the plant (location 2); Figure S3: Impact of plant architecture treatments on cannabinoid concentrations in an apical inflorescence of a bottom branch (location 3); Figure S4: Impact of plant architecture treatments on cannabinoid concentrations in an axillary inflorescence of a top branch (location 4); Figure S5: Impact of plant architecture treatments on cannabinoid concentrations in an axillary inflorescence of a low branch (location 5).

## References

[1] Hanuš L.O., Hod Y. (2020). Terpenes/terpenoids in cannabis: Are they important?. Med. Cannabis Cannabinoids.

[2] Pratt M., Stevens A., Thuku M., Butler C., Skidmore B., Wieland L.S., Clemons M., Kanji S., Hutton B. (2018). Benefits and harms of medical marijuana. Syst. Rev..

[3] Russo E.B. (2019). The case for the entourage effect and conventional breeding of clinical cannabis: No “Strain”, no gain. Front. Plant Sci..

[4] Shiponi S., Bernstein N. (2021). The highs and lows of P supply in medical cannabis: Effects on cannabinoids, the ionome and morpho-physiology. Front. Plant Sci..

[5] Saloner A., Sacks M.M., Bernstein N. (2019). Response of medical cannabis (*Cannabis sativa* L.) genotypes to K supply under long photoperiod. Front. Plant Sci..

[6] Bernstein N., Gorelick J., Zerahia R., Koch S. (2019). Impact of N, P, K, and humic acid supplementation on the chemical profile of medical cannabis (*Cannabis sativa* L.). Front. Plant Sci..

[7] Saloner A., Bernstein N. (2021). Nitrogen supply affects cannabinoid and terpenoid profile in medical cannabis (*Cannabis sativa* L.). Ind. Crop. Prod..

[8] Magagnini G., Grassi G., Kotiranta S. (2018). The effect of light spectrum on the morphology and cannabinoid content of *Cannabis sativa* L.. Med. Cannabis Cannabinoids.

[9] Danziger N., Bernstein N. (2021). Light matters: Effect of light spectra on cannabinoid profile and plant development of medical cannabis (*Cannabis sativa* L.). Ind. Crop. Prod..

[10] Gorelick J., Bernstein N., Chandra S., Lata H., ElSohly M. (2017). Chemical and physical elicitation for enhanced cannabinoid production in cannabis. Cannabis sativa L.—Botany and Biotechnology.

[11] Bernstein N., Gorelick J., Koch S. (2019). Interplay between chemistry and morphology in medical cannabis (*Cannabis sativa* L.). Ind. Crop. Prod..

[12] Bauerle W.L., Bowden J.D., Wang G.G. (2007). The influence of temperature on within-canopy acclimation and variation in leaf photosynthesis: Spatial acclimation to microclimate gradients among climatically divergent *Acer rubrum* L. genotypes. J. Exp. Bot..

[13] Pereira G.E., Gaudillere J.-P., Pieri P., Hilbert G., Maucourt M., Deborde C., Moing A., Rolin D. (2006). Microclimate influence on mineral and metabolic profiles of grape berries. J. Agric. Food Chem..

[14] Wen Y., Zhang Y., Su S., Yang S., Ma L., Zhang L., Wang X. (2019). Effects of tree shape on the microclimate and fruit quality parameters of *Camellia oleifera* abel. Forests.

[15] Bulyaba R., Lenssen A.W. (2019). Nutritional composition of grain legume leaves and the impact of leaf removal on yield. Agrosystems Geosci. Environ..

[16] Hunter J.J., De Villiers O.T., Watts J.E. (1991). The effect of partial defoliation on quality characteristics of *Vitis Vinifera* L. cv. Cabernet Sauvignon grapes 2. skin color, skin sugar, and wine quality. Am. J. Enol. Vitic..

[17] da Silva G.L., Queiroga R.C.F., Pereira F.H.F., de Sousa F.F., da Silva Z.L., Ferreira R.P., de Oliveira O.H. (2019). Effects of fruit thinning and main stem pruning in melon crops. J. Exp. Agric. Int..

[18] Alsadon A., Wahb-Allah M., Abdel-Razzak H., Ibrahim A. (2013). Effects of pruning systems on growth, fruit yield and quality traits of three greenhouse-grown bell pepper (*Capsicum annuum* L.) cultivars. Aust. J. Crop. Sci..

[19] Ačko D.K., Flajšman M., Trdan S. (2019). Apical bud removal increased seed yield in hemp (*Cannabis sativa* L.). Acta Agric. Scand. Sect. B Soil Plant Sci..

[20] Danziger N., Bernstein N. (2021). Plant architecture manipulation increases cannabinoid standardization in ‘drug-type’ medical cannabis. Ind. Crop. Prod..

[21] Russell G.L., Jarvis P.G., Monteith J.L. (1989). Absorption of radiation by canopies and stand growth. Plant Canopies Growth Form Funct..

[22] Hawley D., Graham T., Stasiak M., Dixon M. (2018). Improving Cannabis bud quality and yield with subcanopy lighting. HortScience.

[23] Kasperbauer M.J. (1971). Spectral distribution of light in a tobacco canopy and effects of end-of-day light quality on growth and development. Plant Physiol..

[24] Rameau C., Bertheloot J., Leduc N., Andrieu B., Foucher F., Sakr S. (2015). Multiple pathways regulate shoot branching. Front. Plant Sci..

[25] Katyayini N.U., Rinne P.L.H., Tarkowská D., Strnad M., van der Schoot C. (2020). Dual role of gibberellin in perennial shoot branching: Inhibition and activation. Front. Plant Sci..

[26] Spitzer-Rimon B., Duchin S., Bernstein N., Kamenetsky R. (2019). Architecture and florogenesis in female *Cannabis sativa* plants. Front. Plant Sci..

[27] Liu C.-J., Zhao Y., Zhang K. (2019). Cytokinin transporters: Multisite players in cytokinin homeostasis and signal distribution. Front. Plant Sci..

[28] Kuiper P.J.C., Bierhuizen J.F. (1958). The Effect of some environmental factors on the transpiration of plants under controlled conditions. Environ. Sci..

[29] Ambroszczyk A.M., Cebula S., Sekara A. (2008). The effect of plant pruning on the light conditions and vegetative development of eggplant (*Solanumm elongena* L.) in greenhouse cultivation. Veg. Crop. Res. Bull..

[30] Jung S.-K., Choi H.-S. (2010). Light penetration, growth, and fruit productivity in “Fuji” apple trees trained to four growing systems. Sci. Hortic. (Amsterdam).

[31] Boulard T., Roy J.-C., Pouillard J.-B., Fatnassi H., Grisey A. (2017). Modelling of micrometeorology, canopy transpiration and photosynthesis in a closed greenhouse using computational fluid dynamics. Biosyst. Eng..

[32] Kichah A., Bournet P.-E., Migeon C., Boulard T. (2012). Measurement and CFD simulation of microclimate characteristics and transpiration of an impatiens pot plant crop in a greenhouse. Biosyst. Eng..

[33] Pons T.L., De Jong-Van Berkel Y.E.M. (2004). Species-specific variation in the importance of the spectral quality gradient in canopies as a signal for photosynthetic resource partitioning. Ann. Bot..

[34] Degenhardt F., Stehle F., Kayser O. (2017). The Biosynthesis of Cannabinoids.

[35] Sareer O., Bernstein N., Ahmad S., Umar S. (2016). Genetic, developmental and temporal variability in nitrate accumulation and nitrate reductase activity in the medicinal herb *Andrographis paniculata*. Pedosphere.

[36] Ouzounis T., Rosenqvist E., Ottosen C.-O. (2015). Spectral effects of artificial light on plant physiology and secondary metabolism: A review. HortScience.

[37] Akula R., Ravishankar G.A. (2011). Influence of abiotic stress signals on secondary metabolites in plants. Plant Signal. Behav..

[38] Fischer R., Nitzan N., Chaimovitsh D., Rubin B., Dudai N. (2011). Variation in essential oil composition within individual leaves of sweet basil (*Ocimum basilicum* L.) is more affected by leaf position than by leaf age. J. Agric. Food Chem..

[39] Namdar D., Charuvi D., Ajjampura V., Mazuz M., Ion A., Kamara I., Koltai H. (2019). LED lighting affects the composition and biological activity of *Cannabis sativa* secondary metabolites. Ind. Crop. Prod..

[40] Ma Z., Li S., Zhang M., Jiang S., Xiao Y. (2010). Light intensity affects growth, photosynthetic capability, and total flavonoid accumulation of Anoectochilus plants. HortScience.

[41] Dou H., Niu G., Gu M., Masabni J.G. (2018). Responses of sweet basil to different daily light integrals in photosynthesis, morphology, yield, and nutritional quality. HortScience.

[42] Pettersen R.I., Torre S., Gislerød H.R. (2010). Effects of intracanopy lighting on photosynthetic characteristics in cucumber. Sci. Hortic. (Amsterdam).

[43] Lu N., Maruo T., Johkan M., Hohjo M., Tsukagoshi S., Ito Y., Ichimura T., Shinohara Y. (2012). Effects of supplemental lighting within the canopy at different developing stages on tomato yield and quality of single-truss tomato plants grown at high density. Environ. Control Biol..

[44] Hovi-Pekkanen T., Tahvonen R. (2008). Effects of interlighting on yield and external fruit quality in year-round cultivated cucumber. Sci. Hortic. (Amsterdam).

[45] Crini G., Lichtfouse E., Chanet G., Morin-Crini N. (2020). Applications of hemp in textiles, paper industry, insulation and building materials, horticulture, animal nutrition, food and beverages, nutraceuticals, cosmetics and hygiene, medicine, agrochemistry, energy production and environment: A review. Environ. Chem. Lett..

[46] Raffo A., Mozzanini E., Nicoli S.F., Lupotto E., Cervelli C. (2020). Effect of light intensity and water availability on plant growth, essential oil production and composition in *Rosmarinus officinalis* L.. Eur. Food Res. Technol..

[47] Degani A.V., Dudai N., Bechar A., Vaknin Y. (2016). Shade Effects on leaf production and essential oil content and composition of the novel herb *Eucalyptus citriodora* Hook. J. Essent. Oil-Bear. Plants.

[48] Rezaei M., Razmjoo J., Ehtemam M.H., Karimmojeni H., Zahedi M. (2019). The interaction between shade and drought affects essential oil quantity and quality of Vitex agnus-castus L. leaves and seeds. Ind. Crop. Prod..

[49] Chang X., Alderson P.G., Wright C.J. (2008). Solar irradiance level alters the growth of basil (*Ocimum basilicum* L.) and its content of volatile oils. Environ. Exp. Bot..

