# Supplementary material for: Shape Matters: Plant Architecture Affects Chemical Uniformity in Large-Size Medical Cannabis Plants
Source: Plants (Basel). 2021 Sep 3;10(9):1834. doi: 10.3390/plants10091834 (PMC8468686; doi:10.3390/plants10091834)
Supplement: Supplementary file 1 [file plants-10-01834-s001.zip › plants-1293912-supplementary.pdf]

Supplementary material

Shape matters: plant architecture affects chemical uniformity in large-size medical cannabis plants

Nadav Danziger and Nirit Bernstein *

Institute of Soil water and Environmental Sciences, Volcani Center, 68 HaMaccabim Road
P.O.B 15159 Rishon LeZion, 7505101, Israel

* Correspondence: Dr. Nirit Bernstein: Nirit@agri.gov.il

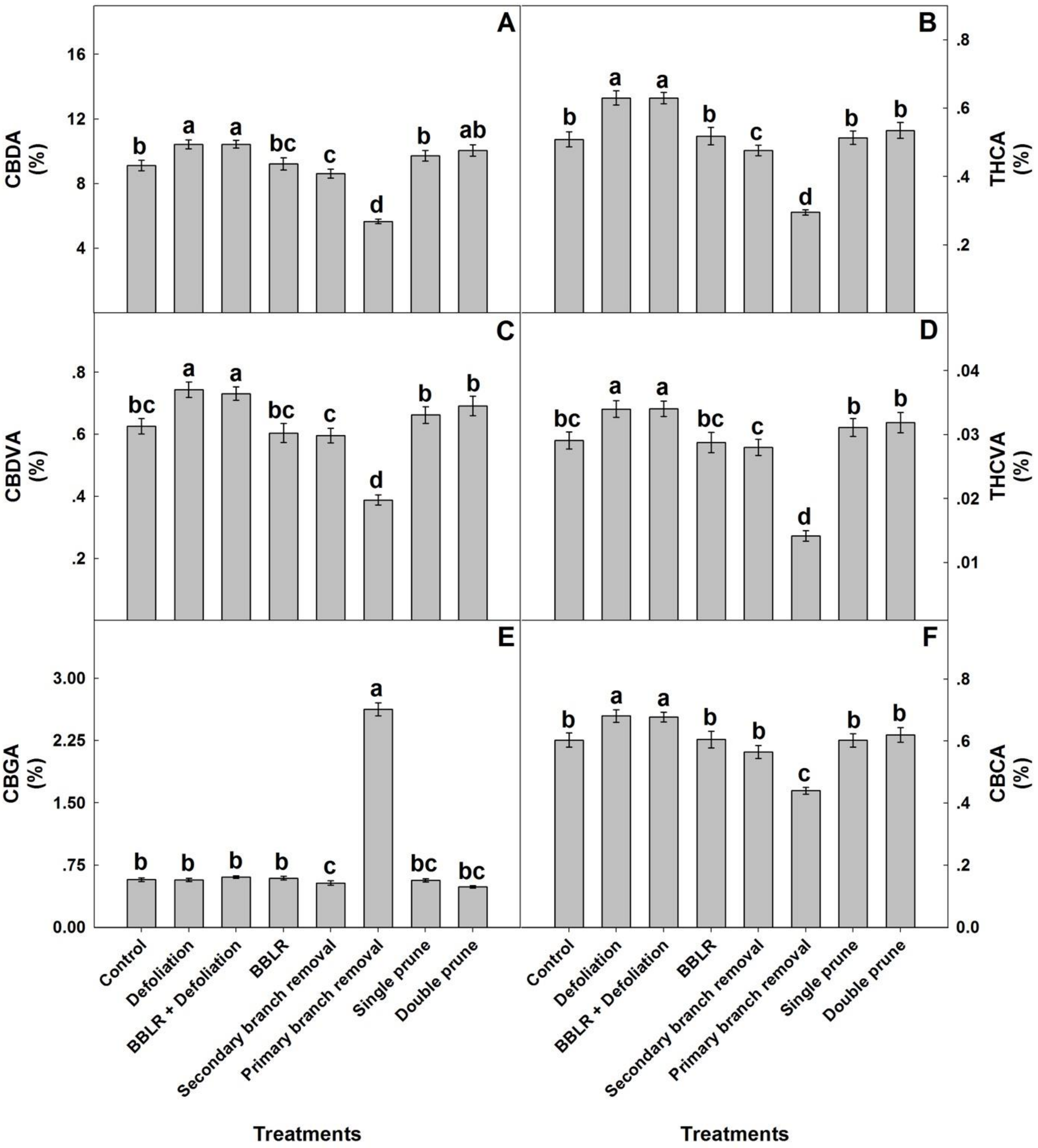

<u>Figure S1</u>. Impact of plant architecture treatments on cannabinoid concentrations in the **apical inflorescence of the main stem (location 1)** of medical cannabis plants CBDA (A), THCA (B), CBDVA (C), THCVA (D), CBGA (E) and CBCA (F). Data are means ± SE (n=6). Lowercase letters above the bars represent significant differences between treatments by Tukey HSD test at α=0.05.

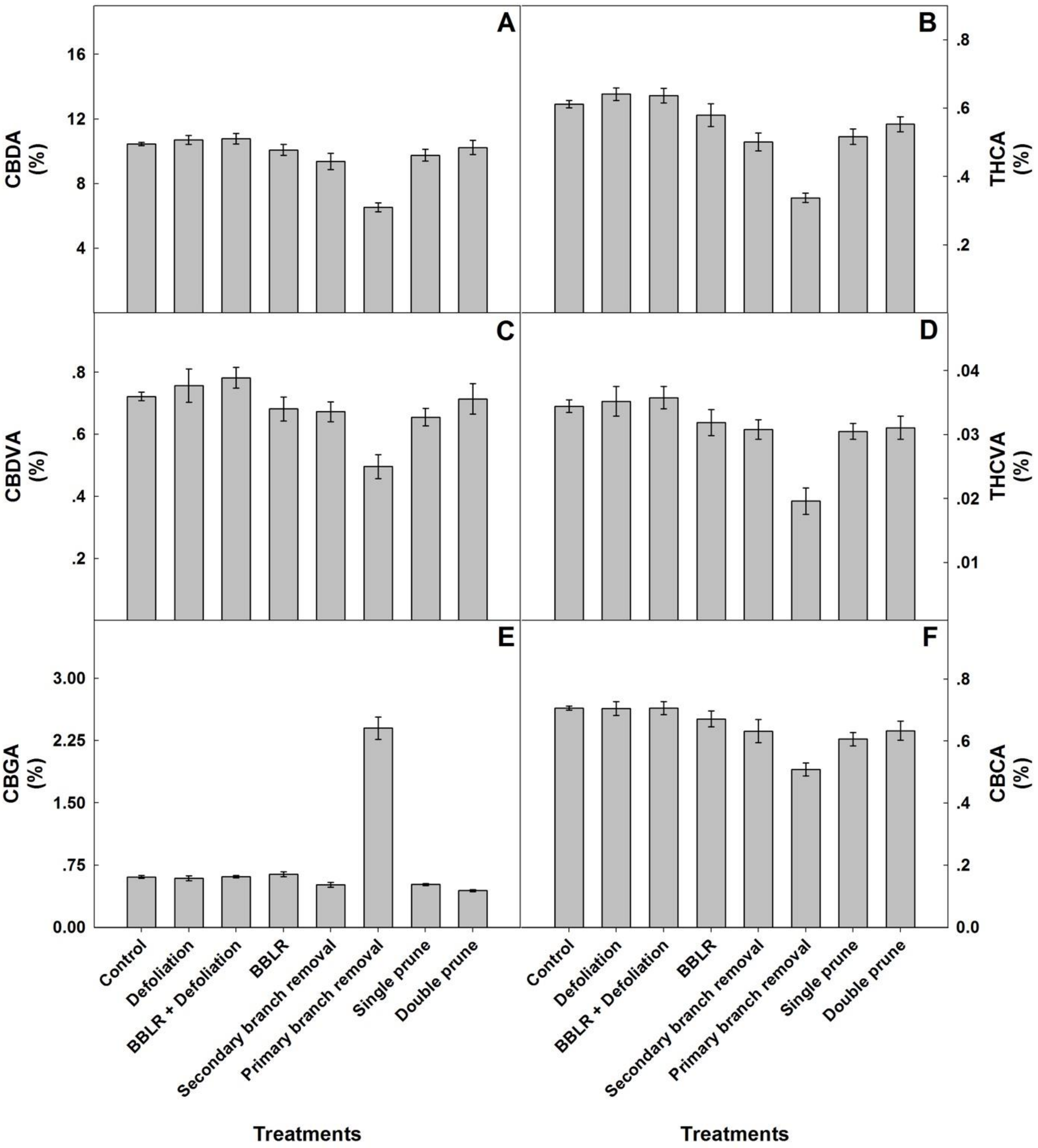

<u>Figure S2</u>. Impact of plant architecture treatments on cannabinoid concentrations at the apical inflorescence from a 1° branch, from the <u>top</u> 1/3 of the plant **(location 2)** of medical cannabis plants CBDA (A), THCA (B), CBDVA (C), THCVA (D), CBGA (E) and CBCA (F). Data are means ± SE (n=6).

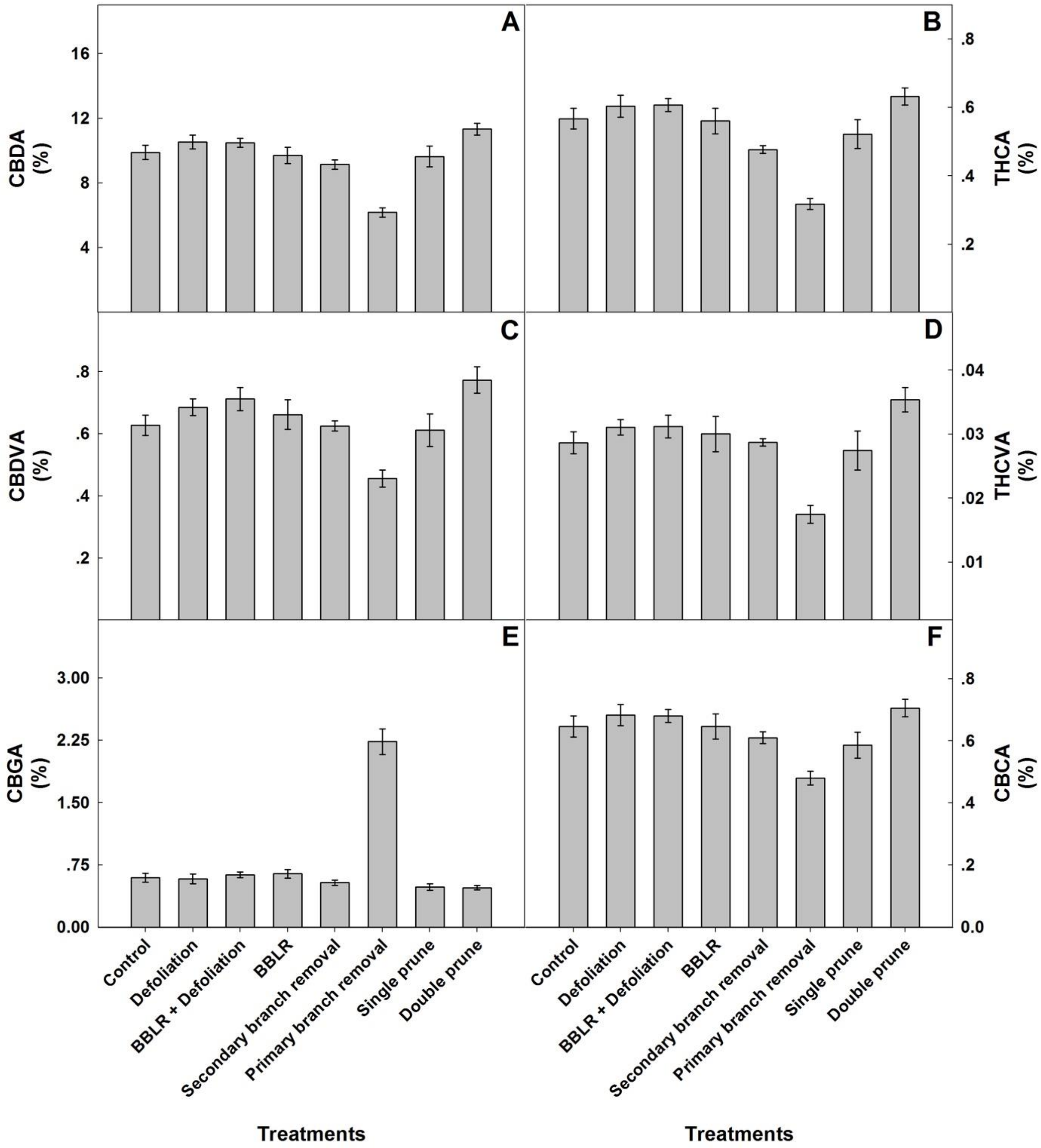

Figure S3. Impact of plant architecture treatments on cannabinoid concentrations in an **apical inflorescence of a** 1° branch from the bottom 1/3 of the plant **(location 3)** of medical cannabis plants CBDA (A), THCA (B), CBDVA (C), THCVA (D), CBGA (E) and CBCA (F). Data are means ± SE (n=6).

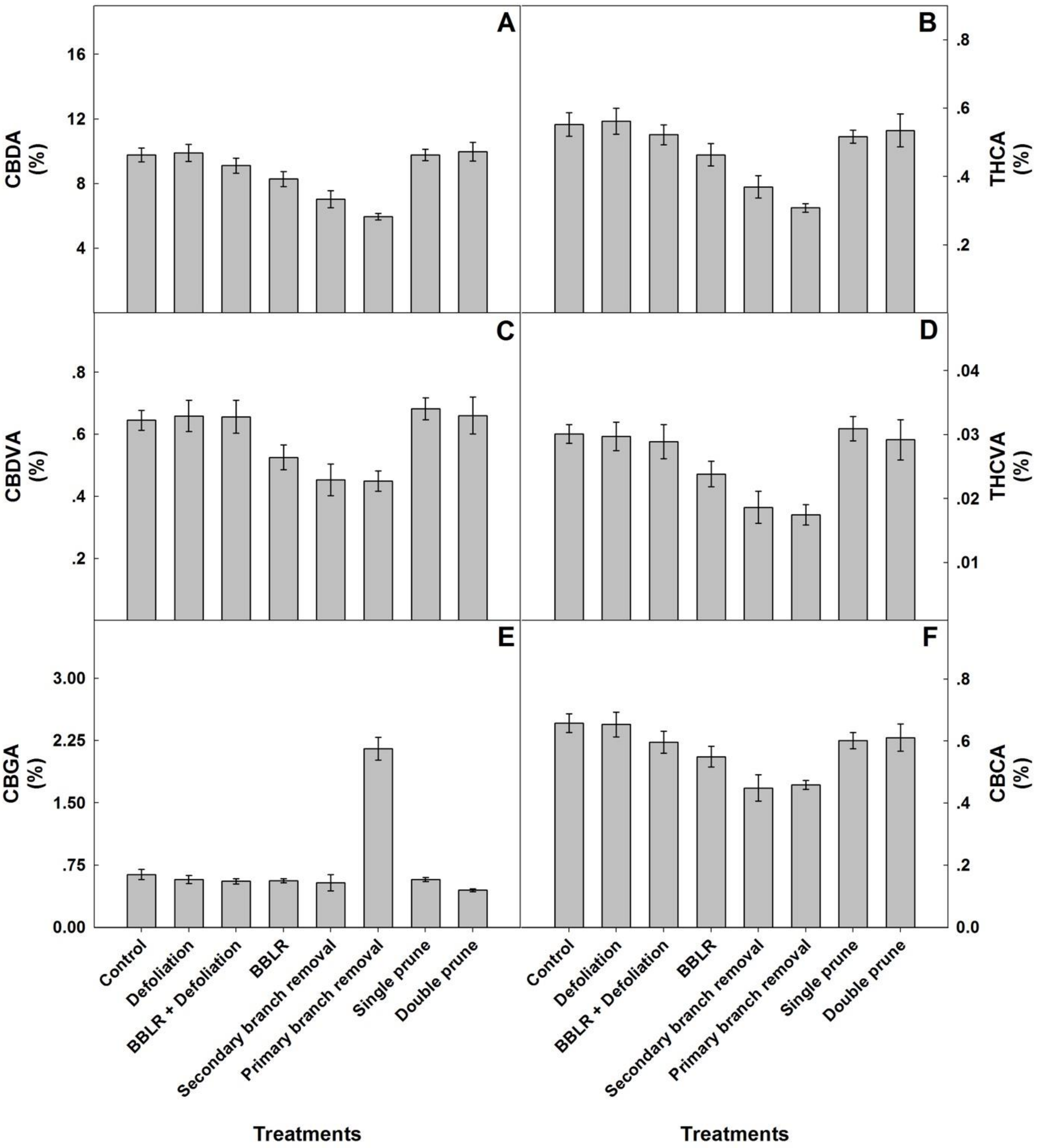

<u>Figure S4</u>. Impact of plant architecture treatments on cannabinoid concentrations in an **axillary inflorescence close to the stem from an upper branch (location 4)** of medical cannabis plants CBDA (A), THCA (B), CBDVA (C), THCVA (D), CBGA (E) and CBCA (F). Data are means ± SE (n=6).

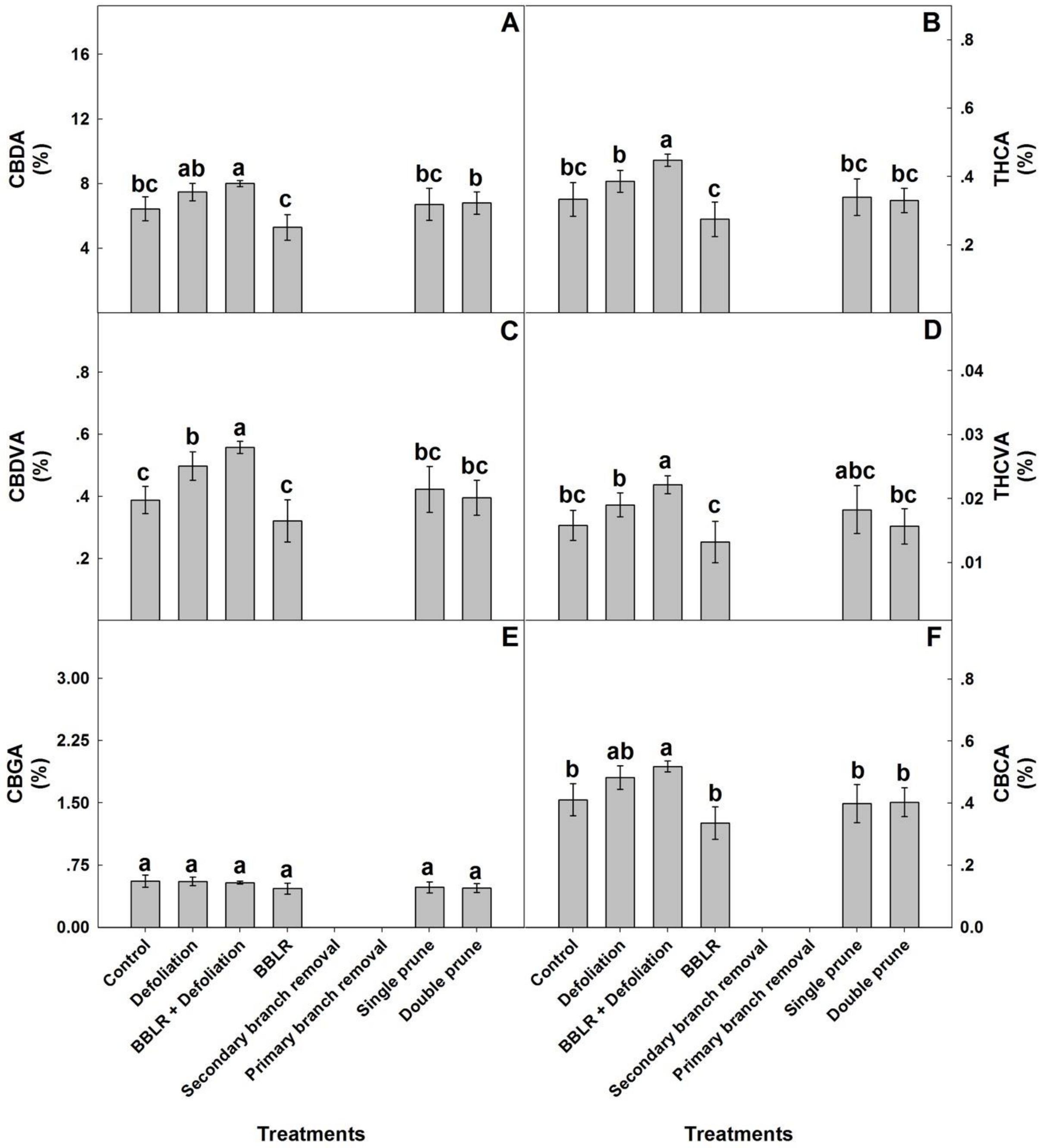

Figure S5. Impact of plant architecture treatments on cannabinoid concentrations in an **axillary inflorescence close to the stem, from the bottom of the plant (location 5)** of medical cannabis plants CBDA (A), THCA (B), CBDVA (C), THCVA (D), CBGA (E) and CBCA (F). Data are means ± SE (n=6). Lowercase letters above the bars represent significant differences between treatments by Tukey HSD test at α=0.05.
